# Supplementary material for: A cross-sectional study of the psychological impact of the COVID-19 pandemic on camped refugees in Ghana
Source: PLoS One. 2022 Nov 30;17(11):e0277515. doi: 10.1371/journal.pone.0277515 (PMC9710794; doi:10.1371/journal.pone.0277515)
Supplement: Attachment — Submitted filename: Response to Editor and Reviewers Comments.docx [file pone.0277515.s001.docx]

**Response to Reviewer’s comments**

PONE-D-22-20247

A cross-sectional study of the psychological impact of the COVID-19 pandemic on camped refugees in Ghana

PLOS ONE

We are grateful to the Editor and Reviewers for providing us with positive feedback and useful suggestions for ‎the revision. The following notes explain how we addressed each comment in the revised ‎manuscript. Please note C1, C2… refer to comments from the Editor and Reviewers and R1, R2… refer to ‎authors response. The corrections are highlighted in yellow shade. ‎

**Journal Requirements**

When submitting your revision, we need you to address these additional requirements.

C1. Please ensure that your manuscript meets PLOS ONE's style requirements, including those for file naming. The PLOS ONE style templates can be found at 
<https://journals.plos.org/plosone/s/file?id=wjVg/PLOSOne_formatting_sample_main_body.pdf> and 
<https://journals.plos.org/plosone/s/file?id=ba62/PLOSOne_formatting_sample_title_authors_affiliations.pdf>.

R1. Thank you for the comment. The style has been revised to meet PLOS ONE’s style requirements.

C2. Please provide additional details regarding participant consent. In the ethics statement in the Methods and online submission information, please ensure that you have specified (1) whether consent was informed and (2) what type you obtained (for instance, written or verbal, and if verbal, how it was documented and witnessed). If your study included minors, state whether you obtained consent from parents or guardians. If the need for consent was waived by the ethics committee, please include this information.

If you are reporting a retrospective study of medical records or archived samples, please ensure that you have discussed whether all data were fully anonymized before you accessed them and/or whether the IRB or ethics committee waived the requirement for informed consent. If patients provided informed written consent to have data from their medical records used in research, please include this information.

R2. Informed consent, explaining the objectives of study and its relevance and assuring respondents of anonymity and confidentiality, was sort before interviews were conducted. Respondents who could read were made to read the informed consent themselves before signing them. For those who could not read, the informed consent was read to them and where necessary interpreted before signing or thumbprinting. Where respondents were below 18 years of age, informed consent was sort from parents/guardians. This has been indicated in the revised manuscript. Please, see page 5, line 20-23 and page 6, line 1 and 2. We have also added the ethical considerations. Please, see page 6, lines 3-13.

C3. We note that you have indicated that data from this study are available upon request. PLOS only allows data to be available upon request if there are legal or ethical restrictions on sharing data publicly. For information on unacceptable data access restrictions, please see <http://journals.plos.org/plosone/s/data-availability#loc-unacceptable-data-access-restrictions>.

In your revised cover letter, please address the following prompts:

a) If there are ethical or legal restrictions on sharing a de-identified data set, please explain them in detail (e.g., data contain potentially identifying or sensitive patient information) and who has imposed them (e.g., an ethics committee). Please also provide contact information for a data access committee, ethics committee, or other institutional body to which data requests may be sent.

b) If there are no restrictions, please upload the minimal anonymized data set necessary to replicate your study findings as either Supporting Information files or to a stable, public repository and provide us with the relevant URLs, DOIs, or accession numbers. Please see <http://www.bmj.com/content/340/bmj.c181.long> for guidelines on how to de-identify and prepare clinical data for publication. For a list of acceptable repositories, please see <http://journals.plos.org/plosone/s/data-availability#loc-recommended-repositories>.

We will update your Data Availability statement on your behalf to reflect the information you provide.

R3. Thank you. The data availability statement has been revised based on your suggestions. Please see 20, lines 17-21.

C4. We note that Figure 1 in your submission contain map/satellite images which may be copyrighted. All PLOS content is published under the Creative Commons Attribution License (CC BY 4.0), which means that the manuscript, images, and Supporting Information files will be freely available online, and any third party is permitted to access, download, copy, distribute, and use these materials in any way, even commercially, with proper attribution. For these reasons, we cannot publish previously copyrighted maps or satellite images created using proprietary data, such as Google software (Google Maps, Street View, and Earth). For more information, see our copyright guidelines: <http://journals.plos.org/plosone/s/licenses-and-copyright>.
 
We require you to either (1) present written permission from the copyright holder to publish these figures specifically under the CC BY 4.0 license, or (2) remove the figures from your submission:
 
a) You may seek permission from the original copyright holder of Figure 1 to publish the content specifically under the CC BY 4.0 license.  
 
We recommend that you contact the original copyright holder with the Content Permission Form (<http://journals.plos.org/plosone/s/file?id=7c09/content-permission-form.pdf>) and the following text:
“I request permission for the open-access journal PLOS ONE to publish XXX under the Creative Commons Attribution License (CCAL) CC BY 4.0 (<http://creativecommons.org/licenses/by/4.0/>). Please be aware that this license allows unrestricted use and distribution, even commercially, by third parties. Please reply and provide explicit written permission to publish XXX under a CC BY license and complete the attached form.”
 
Please upload the completed Content Permission Form or other proof of granted permissions as an "Other" file with your submission.
 
In the figure caption of the copyrighted figure, please include the following text: “Reprinted from [ref] under a CC BY license, with permission from [name of publisher], original copyright [original copyright year].”
 
b) If you are unable to obtain permission from the original copyright holder to publish these figures under the CC BY 4.0 license or if the copyright holder’s requirements are incompatible with the CC BY 4.0 license, please either i) remove the figure or ii) supply a replacement figure that complies with the CC BY 4.0 license. Please check copyright information on all replacement figures and update the figure caption with source information. If applicable, please specify in the figure caption text when a figure is similar but not identical to the original image and is therefore for illustrative purposes only.
The following resources for replacing copyrighted map figures may be helpful:
 
USGS National Map Viewer (public domain): <http://viewer.nationalmap.gov/viewer/>
The Gateway to Astronaut Photography of Earth (public domain): <http://eol.jsc.nasa.gov/sseop/clickmap/>
Maps at the CIA (public domain): <https://www.cia.gov/library/publications/the-world-factbook/index.html> and <https://www.cia.gov/library/publications/cia-maps-publications/index.html>
NASA Earth Observatory (public domain): <http://earthobservatory.nasa.gov/>
Landsat: <http://landsat.visibleearth.nasa.gov/>
USGS EROS (Earth Resources Observatory and Science (EROS) Center) (public domain): [http://eros.usgs.gov/#](http://eros.usgs.gov/)
Natural Earth (public domain): <http://www.naturalearthdata.com/>

R4. Thank you for the clarification. We have removed the figure from the revised manuscript.

C5. Please review your reference list to ensure that it is complete and correct. If you have cited papers that have been retracted, please include the rationale for doing so in the manuscript text, or remove these references and replace them with relevant current references. Any changes to the reference list should be mentioned in the rebuttal letter that accompanies your revised manuscript. If you need to cite a retracted article, indicate the article’s retracted status in the References list and also include a citation and full reference for the retraction notice.

R5. We have checked all the references to ensure that they are cited in the manuscript. Additional references included at the revision stage are indicated in this document. Please, see pages 5 and 6 of this document.

**Comments from Reviewer 1**

Reviewer #1: Dear authors, Thank you for your valuable work and efforts. I have few comments.

C6. Please, Add study design in obvious title and illustrate it.

R6. Thank you for the comment. The study design was cross-sectional. This has been included in the revised title. Please, see page 1, line 1.

C7. Also, Add sample type

R7. A two-stage sampling approach was used to select respondents. At the first stage, a systematic sampling approach was used to select housing units. At the second stage, where more than one eligible respondent was identified in a housing unit, simple random sampling approach was used to select one respondent. This has been clarified in the revised manuscript. Please, see page 6, line 21, 24-25 and page 7, lines 6-7.

C8. Add ethical considerations

R8. Thank you. We have included the ethical considerations in the revised manuscript. Please, see page 5, lines 20-23 and page 6, line 1-13.

**Comments from Reviewer 2**

Reviewer #2: This is an interesting work. This is exciting and novel work. Studying the impact of the COVID-19 pandemic on a particularly vulnerable population becomes highly relevant. The manuscript is generally well written and methodologically meets sufficient quality criteria to be published. However, I suggest reinforcing some points to improve its quality.

C8. It is recommended to analyze the statistical differences between refugee camps.

R8. Thank you for the comment. We have analysed the differences between the camps and included it in the revised manuscript. The results showed that the camp effects were not statistically significantly different for both the bivariate (Please, see highlighted section of Table 4 and page 11, line 3) and multivariate analysis (since they were not significant, they were not included in Table 5). The camp of residence is also included as a covariate. See page 8, line 22. These are also included among the non-significant covariates in the Discussions. See page 15, line 20.

C9. Although the aim of the study focuses on refugee camps, the findings indicate that the characteristics related to the camps do not have the expected statistical relevance. Thus, the results may reflect vulnerable populations, regardless of their area of location. It is therefore suggested to discuss in depth whether the reported characteristics may represent other disadvantaged groups or present more evidence about why these data only represent individuals in refugee camps.

R9. Thank you for the comment. We noticed that the statement was not clear enough. Since most of the background characteristics were not statistically significant, we intended to imply that the psychological distress of the COVID-19 is not limited to camped refugees of particular background characteristics, for example, by educational attainment, country of origin, sex, etc…but it is a problem in the general population of camped refugees. It is also important to note that, since the data focused on camped refugees we cannot infer to reflect all vulnerable populations. The statement has been modified in the revised manuscript. Please, see page 19, lines 3-7.

C10. Explain in greater detail the findings presented in Table 5, models 1 and 2, regarding the categories that are not significant for the variables (age of the head of the household 40 and over, three or more sources of information on COVID, and marital status. Discuss these findings in the corresponding section.

R10. Thank you. We have provided detailed discussions of the categories that were not significant for the variables age of the household head, number of sources of information and marital status. Please, see page 16, lines 2 – 17 and page 17, lines 3 – 14. The references below have been included in the revised manuscript to support the discussions.

58.	Baird, S., et al., *Intersecting disadvantages for married adolescents: Life after marriage pre-and post-Covid-19 in contexts of displacement.* Journal of Adolescent Health, 2022. **70**(3): p. S86-S96.

59.	Jace, C.E. and C.A. Makridis, *Does marriage protect mental health? Evidence from the COVID‐19 pandemic.* Social Science Quarterly, 2021. **102**(6): p. 2499-2515.

60.	Ahituv, A. and R.I. Lerman, *How do marital status, work effort, and wage rates interact?* Demography, 2007. **44**(3): p. 623-647.

61.	Glenn, N.D. and C.N. Weaver, *The contribution of marital happiness to global happiness.* Journal of Marriage and the Family, 1981: p. 161-168.

62.	Gove, W.R., M. Hughes, and C.B. Style, *Does marriage have positive effects on the psychological well-being of the individual?* Journal of health and social behavior, 1983: p. 122-131.

63.	Kiecolt-Glaser, J.K. and T.L. Newton, *Marriage and health: his and hers.* Psychological bulletin, 2001. **127**(4): p. 472.

64.	Lillard, L.A. and L.J. Waite, *'Til death do us part: Marital disruption and mortality.* American journal of sociology, 1995. **100**(5): p. 1131-1156.

65.	Islam, M.M. and M. Alharthi, *Impact of COVID-19 on the Quality of Life of Households in Saudi Arabia.* International Journal of Environmental Research and Public Health, 2022. **19**(3): p. 1538.

71.	Alrasheed, M., S. Alrasheed, and A.S. Alqahtani, *Impact of Social Media Exposure on Risk Perceptions, Mental Health Outcomes, and Preventive Behaviors during the COVID-19 Pandemic in Saudi Arabia.* Saudi Journal of Health Systems Research: p. 1-7.

72.	Garfin, D.R., R.C. Silver, and E.A. Holman, *The novel coronavirus (COVID-2019) outbreak: Amplification of public health consequences by media exposure.* Health psychology, 2020. **39**(5): p. 355.

73.	Han, R., J. Xu, and D. Pan, *How Media Exposure, Media Trust, and Media Bias Perception Influence Public Evaluation of COVID-19 Pandemic in International Metropolises.* International Journal of Environmental Research and Public Health, 2022. **19**(7): p. 3942.

74.	Purvis, R.S., et al., *Perceptions of adult Arkansans regarding trusted sources of information about the COVID-19 pandemic.* BMC Public Health, 2021. **21**(1): p. 1-9.
