# Supplementary material for: A cross-sectional study of the psychological impact of the COVID-19 pandemic on camped refugees in Ghana
Source: PLoS One. 2022 Nov 30;17(11):e0277515. doi: 10.1371/journal.pone.0277515 (PMC9710794; doi:10.1371/journal.pone.0277515)
Supplement: Attachment — Submitted filename: Response to Editor and Reviewers Comments.docx [file pone.0277515.s002.docx]

**Response to Reviewer’s comments**

PONE-D-22-20247R1

A cross-sectional study of the psychological impact of the COVID-19 pandemic on camped refugees in Ghana

PLOS ONE

We are once again grateful to the Editor and Reviewers for their comments and suggestions. The following notes explain how we addressed the comments in the revised ‎manuscript. Please note C1, C2… refer to comments from the Editor and Reviewers and R1, R2… refer to ‎authors response. The corrections are highlighted in yellow shade. ‎

**Journal Requirements**

C1. Please review your reference list to ensure that it is complete and correct. If you have cited papers that have been retracted, please include the rationale for doing so in the manuscript text, or remove these references and replace them with relevant current references. Any changes to the reference list should be mentioned in the rebuttal letter that accompanies your revised manuscript. If you need to cite a retracted article, indicate the article’s retracted status in the References list and also include a citation and full reference for the retraction notice

R1. Thank you, We have checked the reference list and confirm that they are complete and correct. To address C2, the reference below was added to support the discussion. Please, see page 9, lines 16 – 17 and page 26, lines 4 – 5.

46.	Kim, H.-Y., Statistical notes for clinical researchers: Sample size calculation 2. Comparison of two independent proportions. Restorative Dentistry & Endodontics, 2016. 41(2): p. 154-156.

**Additional Editor Comments**

C2. Add the comparison between both groups for each variable presented in table four. Ensure to add a column with the correspondent significance level.

Thank you for the suggestion. The suggested analysis has been included in the revised manuscript. Please see pages 35 and 36, highlighted section of Table 4. Please, note that Fisher's Exact Test was used to test for significant differences where cell sample size were small (less than five). The reference below has been included to support the decision. Please, see page 9, lines 16 – 17. The results are discussed in the highlighted sections of pages 11 – 13.

46.	Kim, H.-Y., Statistical notes for clinical researchers: Sample size calculation 2. Comparison of two independent proportions. Restorative Dentistry & Endodontics, 2016. 41(2): p. 154-156.
